# FGFR1 is an independent prognostic factor and can be regulated by miR-497 in gastric cancer progression

**DOI:** 10.1590/1414-431X20187816

**Published:** 2018-11-23

**Authors:** Gang Xie, Qi Ke, Yu Zu Ji, An-qun Wang, Meng Jing, li-li Zou

**Affiliations:** Department of Pathology, Mianyang Central Hospital, Mianyang, Sichuan Province, China

**Keywords:** FGFR1, miR-497, Apoptosis, Post-transcriptional, Gastric cancer

## Abstract

Fibroblast growth factor receptor 1 (FGFR1) has been reported in gastric cancer to be a prognostic factor. However, miR-497-targeted FGFR1 has not been explored in the carcinogenesis of gastric cancer. The present study intended to revalidate the prognostic significance of FGFR1 in patients with gastric cancer, and the mechanism of miR-497-regulated FGFR1 was investigated in gastric cancer cell proliferation and apoptosis. The messenger RNA (mRNA) and protein levels were assayed by RT-qPCR and western blotting, respectively. The targeted genes were predicted by a bioinformatics algorithm and confirmed by a dual luciferase reporter assay. Cell proliferation was analyzed by CCK-8 assay. Annexin V-FITC/PI staining was used to evaluate the apoptosis in AGS and SGC-7901 cells. FGFR1 was frequently up-regulated in gastric cancer tissues and associated with poor overall survival in patients with gastric cancer. Interestingly, FGFR1 loss-of-function resulted in a significant growth inhibition and apoptosis in AGS and SGC-7901 cells. In addition, we found that miR-497 was inhibited in gastric cancer tissues and cell lines, while overexpression of miR-497 could suppress proliferation and induce apoptosis in AGS and SGC-7901 cells. Importantly, bioinformatics analysis and experimental data suggested that FGFR1 was a direct target of miR-497, which could inhibit FGFR1 expression when transfected with miR-497 mimics. Furthermore, we found that overexpression of FGFR1 reversed the growth inhibition and apoptosis of miR-497 mimics in AGS and SGC-7901 cells. These findings suggested that overexpression of miR-497 inhibited proliferation and induced apoptosis in gastric cancer through the suppression of FGFR1.

## Introduction

The fibroblast growth factor receptor (FGFR) family is composed of four members, FGFR1/2/3/4, which are activated by interacting with fibroblast growth factors (FGFs), resulting in the activation of downstream signaling pathways including mitogen-activated protein kinase signal transducer and activator of transcription and PI3K/Akt/mTOR signaling (1,2). FGFR1 belongs to receptor tyrosine kinases and possesses various biological functions, such as proliferation, survival, migration, and differentiation (3). FGFR1 is expressed on a number of different cell types including tumor cells (4,5). Intriguingly, overexpression of FGFR1 has been observed in multiple human malignancies (6–8). A meta-analysis and clinical survey suggest that FGFR1 is emerging as a diagnostic and prognostic marker for non-small-cell lung cancer (NSCLC) (9,10). However, its functional contributions have not been completely clarified in gastric cancer.

MicroRNAs (miRNAs) are short, noncoding, single-stranded RNAs (18-25 nucleotides) that have been verified to suppress protein translation by binding to the 3′-untranslated regions of target gene (11). miRNAs function as a novel class of post-transcriptional regulators participating in a variety of biological processes, including angiogenesis, tumor proliferation, and metastasis (12). For example, miR-338-3p inhibits migration and induces apoptosis in gastric cancer cells by blocking protein-tyrosine phosphatase 1B expression (13). miR-148b-3p inhibits gastric cancer metastasis by inhibiting the Dock6/Rac1/Cdc42 axis (14). However, the underlying molecular mechanism of miR-497 in gastric cancer progression by targeting FGFR1 is rarely investigated.

miR-497 belongs to the miR-15 family and negatively regulates angiogenesis in tumors (15). Numerous studies have suggested that miR-497 can serve as a tumor suppressor in a variety of tumors, including renal cell carcinoma (16), NSCLC (17), breast cancer (18), and hepatocellular carcinoma (19). In gastric cancer, lncRNA-GACAT3 and XIST promote cell growth and invasion by negatively regulating miR-497 expression (20,21). Overexpression of miR-497 inhibits gastric cancer cells growth and metastasis *in vitro* and *in vivo* (22).

In the present study, we aimed to evaluate the prognostic significance of FGFR1 in patients with gastric cancer, and the mechanism of miR-497-regulated FGFR1 in gastric cancer cell proliferation and apoptosis.

## Material and Methods

Seventy-four pairs of gastric cancer tissues and adjacent non-tumor tissues were collected from patients who had undergone surgery at the Mianyang Central Hospital (China) between January 2009 and June 2014. All of the patients were recruited according to the histopathological evaluation without radiotherapy or chemotherapy before surgical operation. The tissues were immediately stored in liquid nitrogen after surgery. Informed consent forms were obtained from the patients. This study was approved by the Ethics Committee of the Mianyang Central Hospital (China).

### Cell culture

Normal human GES-1 cell line and five gastric cancer cell lines (MGC-803, AGS, HGC-27, SGC-7901, and BGC-823) were purchased from the Cell Bank of Type Culture Collection of Chinese Academy of Sciences (China). Cells were cultured in Dulbecco's modified Eagle's medium (DMEM; Invitrogen, USA) with 5% fetal bovine serum (Thermo Scientific HyClone, China), in a humidified incubator (Thermo, USA) with 5% CO^2^, 95% air.

### Cell transfection and plasmid constructs

miR-NC and miR-497 were synthesized by RiboBio (China). Cells were seeded in 6-well plates and transfected with miR-NC and miR-497 using Lipofectamine 2000 (Invitrogen; Thermo Fisher Scientifc, Inc., USA) for 48 h at 37°C according to the manufacturer's protocol.

Short hairpin RNA (shRNA) was constructed to specifically target FGFR1 by shRNA design tools (http://rnaidesigner.thermofisher.com/rnaiexpress/). Using BLAST (http://blast.ncbi.nlm.nih.gov/Blast.cgi), we verified that the designed shRNA targeted only the FGFR1. Sh-FGFR1 and sh-NC were synthesized by GenePharma (China).

A mammalian expression plasmid (pReceiver-M02-ERBB3) designed to specially express the full-length open reading frame of human FGFR1 without miR-497 responsive 3′-UTR was purchased from GeneCopoeia (USA). An empty plasmid served as a negative control. Overexpressed FGFR1 plasmid (vector-FGFR1) and control (vector-NC) were transfected into cells using Lipofectamine 2000 (Invitrogen; Thermo Fisher Scientifc, Inc.) for 48 h at 37°C according to the manufacturer's protocol.

### CCK-8 assay

The cell viability of AGS and SGC-7901 cells (1×10^4^) was detected using a CCK-8 assay kit (Japan). The absorbance was measured at 450 nm using a SpectraMax M5 plate reader (Molecular Devices, USA). The CCK-8 proliferation assay was performed as previously described (23).

### Flow cytometry for apoptosis

AGS and SGC-7901 cells were incubated with different conditions for 48 h. Annexin V-FITC/PI kit (Becton, Dickinson and Company, USA) was used to stain cells for 15 min, and then cell apoptosis assay was performed by flow cytometry assay (FACScan, BD Biosciences, USA) and analyzed by CELL Quest 3.0 software (BD Biosciences).

### Luciferase reporter gene assay

The putative binding sites between miR-497 and FGFR1 were obtained using the online prediction softwares Targetscan (http:/www.targetscan.org) and miRanda (http:/www.microrna.org). The wild-type (WT) and mutant-type (MUT) 3′-UTR of FGFR1 (0.5 μg) were inserted into the multiple cloning sites of the luciferase expressing pMIR-REPORT vector (Ambion; Thermo Fisher Scientific, Inc.) and co-transfected with miR-NC or miR-497 (100 nM), and then luciferase activity was evaluated with luciferase reporter assay kit (Beyotime Institute of Biotechnology, China) according to the manufacturer's protocol.

### Immunohistochemical staining

The paraffin-embedded tumor tissues were cut into 3-μm sections and mounted on glass slides for staining with immunoperoxidase, and the procedures of immunohistochemical staining of FGFR1 (Cat. No. ab59194; dilution: 1:50; Abcam, UK) were performed as previously described (24). The slides were visualized under a microscope (Leica DM 2500; Leica, Germany). Image Pro-Plus 6 software (Media Cybernetics, Inc., USA) was used for the analysis of the integrated optical density in the tumor tissues and corresponding adjacent normal tissues of immunohistochemical positive staining.

### Reverse transcription-quantitative polymerase chain reaction (RT-qPCR)

#### miRNA RT-qPCR

The total RNA was isolated using RNAiso (Takara, China). miRNA was subsequently reverse-transcribed to cDNA using the miRNA-specific stem-loop reverse-transcription primer (Ribobio, China). The relative expression levels of miRNA were calculated using the 2^-ΔΔCt^ method (25) and normalized to the internal control U6, using miRNA-specific primers (RiboBio, China). The reaction conditions were performed according to the instructions from Ribobio Co., Ltd with SYBR Green qPCR Mix (BioRad, USA).

#### mRNA RT-qPCR

The cDNA was synthesized by reverse transcription reactions with 2 μg of total RNA using moloney murine leukemia virus reverse transcriptase (Invitrogen; Thermo Fisher Scientific, Inc.) according to the manufacturer's protocol. RT-qPCR was performed by Applied Biosystems 7300 Real-Time PCR System (Thermo Fisher Scientific, Inc.) with the TaqMan Universal PCR Master Mix (Thermo Fisher Scientific, Inc.). The relative expression levels of mRNA were calculated using the 2^-ΔΔCt^ method (25) and normalized to glyceraldehyde 3-phosphate dehydrogenase (GAPDH). The primers were used as follows: FGFR1: forward primer 5'-CACATCGAGGTGAACGGGAGTAAG-3′ and reverse primer 5′-CGCATCCTCAAAGGAGACATTCC-3′; GAPDH: forward primer 5′-GCACCGTCAAGCTGAGAAC-3′ and reverse primer 5′-TGGTGAAGACGCCAGTGGA-3′.

### Western blotting

Proteins were extracted with radio immunoprecipitation assay (RIPA) buffer (Cat. No. P0013B; Beyotime Institute of Biotechnology, China). Fifty micrograms of protein for each sample was separated on a 10% SDS-PAGE gel and transferred to nitrocellulose membranes (BioRad Laboratories, Inc., USA). After blocking with 5% non-fat dry milk at room temperature for 2 h, the membranes were incubated with the primary antibody of FGFR1 (Cat. No. ab59194; dilution: 1: 2,000; Abcam, UK) at room temperature for 2 h. Following three washes with Tris-HCL-buffered saline with Tween-20 (TBST), the membranes were incubated with the appropriate horseradish peroxidase-conjugated secondary antibody (cat.no: sc-516102; dilution: 1:10,000; Santa Cruz Biotechnology, USA) at room temperature for 2 h and visualized by chemiluminescence (Thermo Fisher Scientific, Inc.). β-actin (Cat. No. sc-130065; 1: 2,000; Santa Cruz Biotechnology) was used as the control antibody. Signals were analyzed with Quantity One® software version 4.5 (Bio Rad Laboratories, Inc.).

### Statistical analysis

Data are reported as means±SE. Statistical analysis was performed using IBM SPSS Statistics Version 19.0 (USA) and GraphPad Prism Version 7.0 (USA). Student's *t*-test was used to analyze two-group differences. Inter-group differences were analyzed by one-way analysis of variance, followed by a post-hoc Tukey's test for multiple comparisons. Overall survival was calculated using the Kaplan-Meier method with the log-rank test applied for comparison. Spearman's rank analysis was used to identify the correlation between the expression levels of miR-497 and FGFR1 gastric cancer. P values less than 0.05 were considered to indicate a statistically significant difference.

## Results

### FGFR1 was up-regulated in gastric cancer and associated with poor prognosis

**Figure 1.**
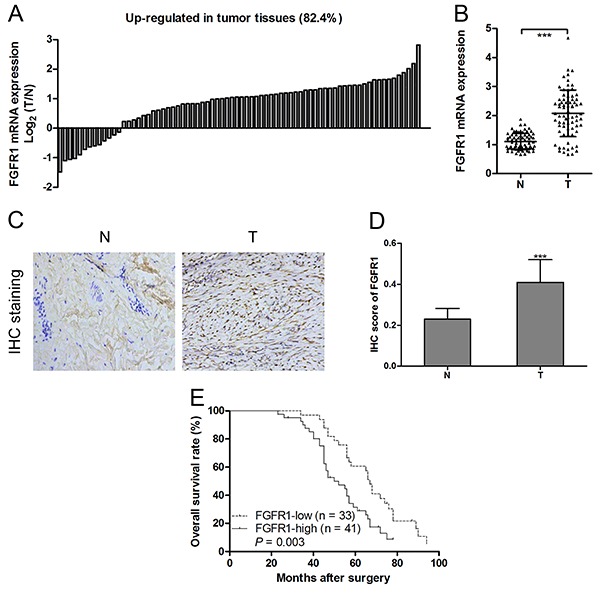
Expression of fibroblast growth factor receptor 1 (FGFR1) measured by RT-qPCR (*A* and *B*) and immunohistochemical (IHC) staining (*C* and *D*; magnification 100×, 50 μm) in 74 pairs of tumor (T) tissues and adjacent non-tumorous (N) tissues from gastric cancer patients. Kaplan-Meier survival curve was used to evaluate whether FGFR1 expression levels were associated with overall survival in patients with gastric cancer (*E*). In *D*, data are reported as means±SE. ***P<0.001 (*t*-test).

FGFR1 signaling plays a crucial role in maintaining cancer properties, such as cell survival, angiogenesis, and metastasis, in a variety of cancers (8,26). Several studies have reported that FGFR1 is overexpressed in gastric cancer tissues and may be considered as a prognostic factor (7,27,28). Consistent with these results, our findings demonstrated that the expression of FGFR1 was increased in 82.4% (63/74) of patients with gastric cancer (Figure 1A). In addition, the mRNA levels of FGFR1 were significantly up-regulated in the gastric cancer tissues compared to the corresponding adjacent normal tissues (Figure 1B). The immunohistochemical staining analysis also showed a significant increase in the levels of FGFR1 in tumor tissues compared with normal tissues (Figure 1C and D). In addition, the association between FGFR1 mRNA expression and overall survival prognosis was evaluated by the Kaplan-Meier survival curve in patients with gastric cancer. Patients were segregated into a high expression group and a low expression group according to the fold-change of FGFR1 in tumor tissue to normal tissue more than 2-fold. The findings showed that patients with high mRNA expression of FGFR1 resulted in a significantly poorer prognosis compared with patients with a low level of FGFR1 (Figure 1E). We also found that high expression of FGFR1 was correlated with TNM stages, lymph nodes metastasis, and distant metastasis or recurrence, but not correlated with gender, age, and tumor size (Table 1).

**Table 1:** Correlation between clinicopathological factors and fibroblast growth factor receptor 1 (FGFR1) expression levels in gastric cancer patients.

Variable	n	FGFR1-Low (n = 33)	FGFR1-High (n = 41)	P value
Gender				0.974
Male	45	20	25	
Female	29	13	16	
Age (years)				0.980
<60	36	16	20	
≧60	38	17	21	
Tumor size (cm)				0.284
≤3	42	21	21	
>3	32	12	20	
TNM stages				0.013
I-II	33	20	13	
III-IV	41	13	28	
Distant metastasis or recurrence				<0.001
Negative	38	25	13	
Positive	36	8	28	
Lymph nodes metastasis				0.001
Negative	35	23	12	
Positive	39	10	29	

TNM: tumor, node, metastasis.

### FGFR1 knockout inhibited proliferation and induced apoptosis in gastric cancer cells

**Figure 2.**
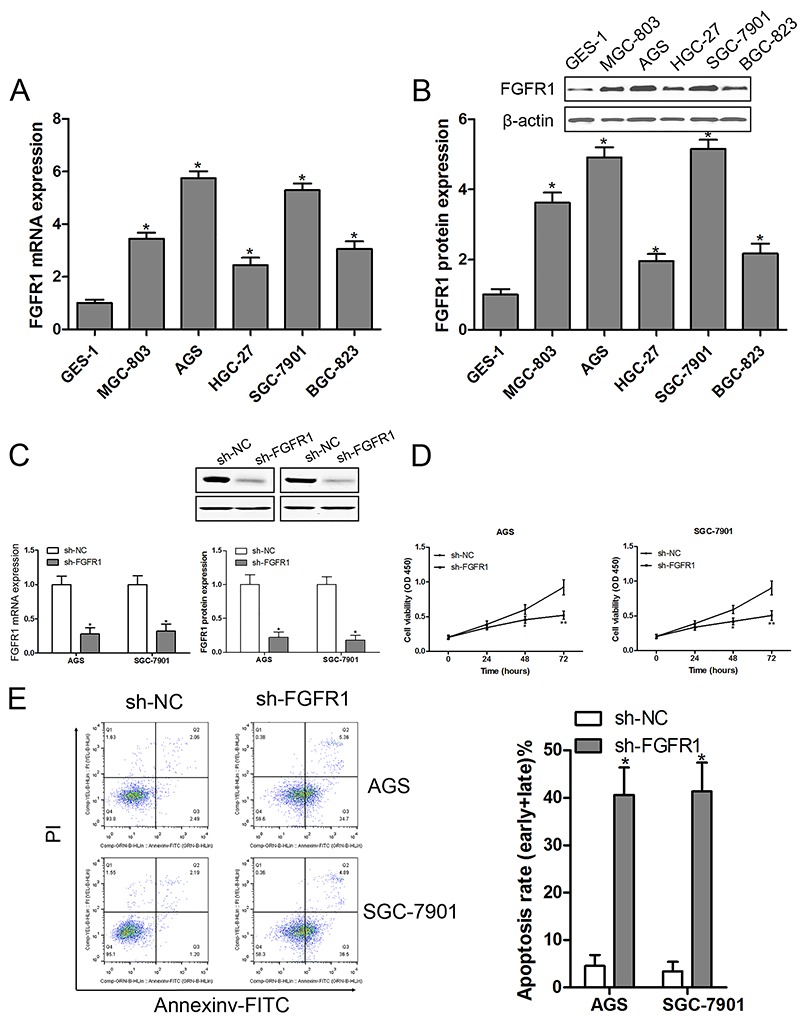
mRNA (*A*) and protein (*B*) expression of fibroblast growth factor receptor 1 (FGFR1) in normal human GES-1 cells and five gastric cancer cell lines measured by RT-qPCR and western blotting, respectively. After transfection with shRNA to inhibit FGFR1 expression, the mRNA and protein expression of FGFR1 were measured by RT-PCR and western blotting, respectively (*C*). Cell viability was measured by CCK-8 assay (*D*), and cell apoptosis by flow cytometry in AGS and SGC-7901 cells (*E*). Data are reported as means±SE for n=3 in each group. *P<0.05, **P<0.01 compared to control (*t*-test or ANOVA).

FGFR1 is implicated in the carcinogenesis of gastric cancer, and FGFR1 inhibitor, L16H50, and L6123 can suppress cell proliferation and migration in gastric cancer cells (6,29). In our study, shRNA was designed to inhibit FGFR1 expression by post-transcriptional gene silencing to explore the functions of FGFR1 in gastric cancer cell proliferation and apoptosis. We found that FGFR1 mRNA and protein levels were significantly up-regulated in all of the gastric cancer cell lines compared with normal GES-1 cells (Figure 2A and B). Intriguingly, the mRNA and protein levels of FGFR1 were markedly higher in AGS and SGC-7901 cells than in MGC-803, HGC-27, and BGC-823 (Figure 2A and B). Hence, AGS and SGC-7901 cells were used on our further experiments. Subsequently, sh-NC or sh-FGFR1 was transfected into AGS and SGC-7901 cells. The results demonstrated that both mRNA and protein expression of FGFR1 were significantly declined with sh-FGFR1 transfection compared with sh-NC administration (Figure 2C); the inhibition rate of sh-FGFR1 to FGFR1 expression was 80%. After transfected with sh-NC or sh-FGFR1, the cell proliferation and apoptosis were measured by CCK-8 and annexin V-FITC/PI staining assay, respectively. The results demonstrated that transfection of sh-FGFR1 suppressed proliferation (Figure 2D) and induced apoptosis (Figure 2E) in AGS and SGC-7901 cells.

### Overexpression of miR-497 inhibited proliferation and induced apoptosis in gastric cancer cells

**Figure 3.**
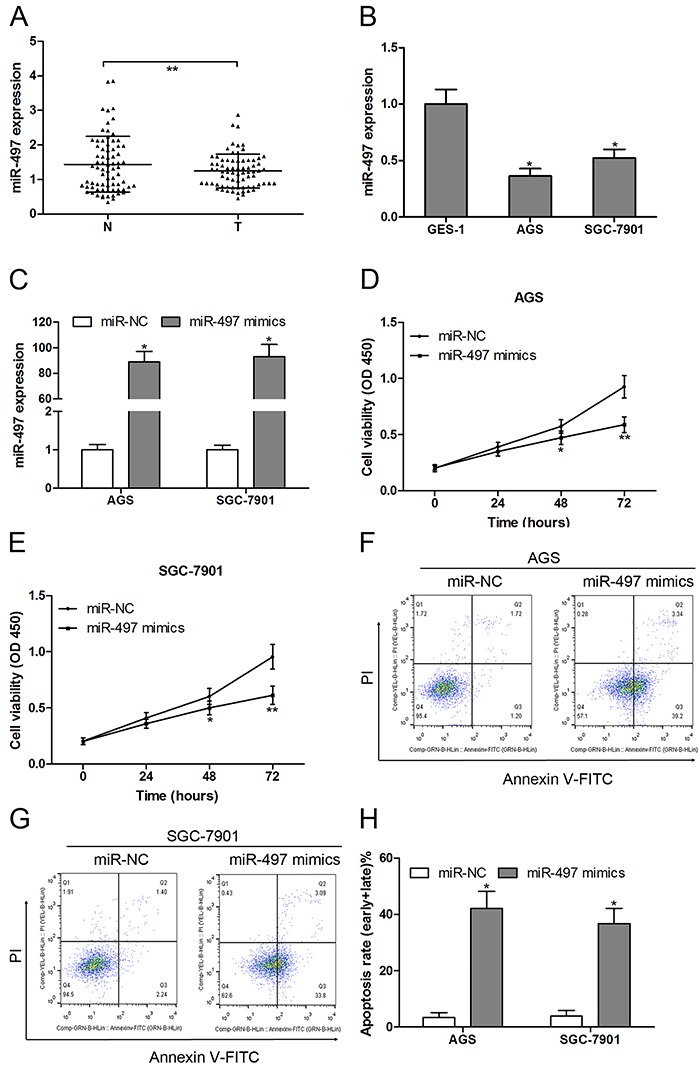
Expression of miR-497 in tumor tissues and adjacent non-tumorous tissues (*A*) or AGS and SGC-7901 cells (*B*) measured by RT-qPCR. The expression of miR-497 was measured by RT-qPCR after transfection with miR-497 mimics (*C*). After transfected with miR-497 mimics, cell viability was measured by CCK-8 assay (*D* and *E*), and cell apoptosis was detected by flow cytometry in AGS and SGC-7901 cells (*F*, *G,* and *H*). Data are reported as means±SE for n=3 in each group. *P<0.05, **P<0.01 (*t*-test or ANOVA).

Recent studies show that miR-497 as an oncogene is inhibited in several cancers (18,30). The present study also showed a significant decrease in the levels of miR-497 in gastric cancer tissues and cell lines (Figure 3A and B). To verify the anti-cancer roles of miR-497 in gastric cancer, miR-497 mimics were transfected into AGS and SGC-7901 cells to measure the effect of miR-497 on cell proliferation and apoptosis. First, we found that transfection with miR-497 mimics led to an increase of miR-497 expression in AGS and SGC-7901 cells (Figure 3C). Further investigations showed that overexpressed miR-497 significantly inhibited cell proliferation (Figure 3D and E) and induced apoptosis (Figure 3F, G and H) in AGS and SGC-7901 cells compared with the control group.

### FGFR1 was a direct target of miR-497

**Figure 4.**
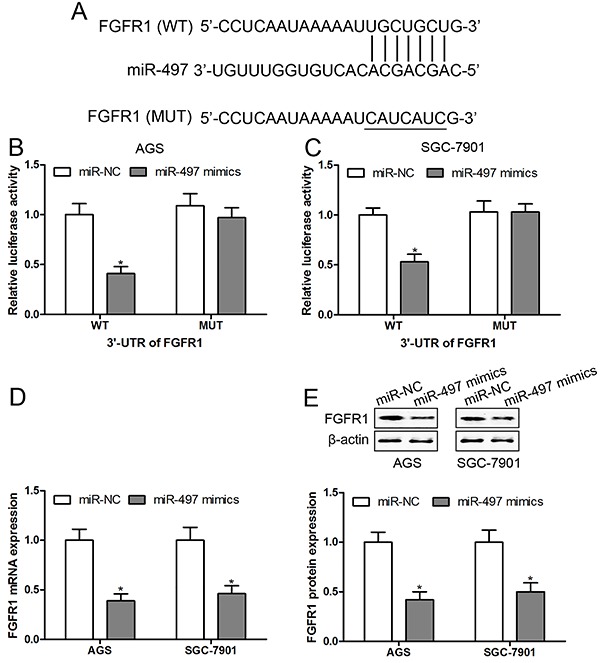
Putative miR-497 binding sites in the 3′-UTR of fibroblast growth factor receptor 1 (FGFR1) predicted by Targetscan and miRanda (*A*). AGS and SGC-7901 cells were co-transfected with the wild type (WT) or mutant (MUT) 3′-UTR of FGFR1 with miR-NC or miR-497 mimics, and the luciferase activity assay was performed after 48 h of transfection (*B* and *C*). The mRNA and protein expression of FGFR1 in AGS and SGC-7901 cells were measured by RT-qPCR and western blotting, respectively (*D* and *E*). Data are reported as means±SE for n=3 in each group. *P<0.05 (*t*-test).

Based on Targetscan and miRanda, we found the 3′-UTR of FGFR1 contained a putative target sequence to bind with miR-497 (Figure 4A). To confirm these, either the WT or MUT sequence of ACE was transfected into the luciferase-reporter plasmids, and then the plasmids were co-transfected with miR-497 mimics into AGS and SGC-7901 cells. We found that miR-497 mimics were able to decrease the luciferase enzyme activity in AGS and SGC-7901 cells transfected with WT 3′-UTR-reporter, while miR-497 mimics had no obvious effect on the luciferase enzyme activity in AGS and SGC-7901 cells transfected with MUT 3′-UTR-reporter (Figure 4B and C). Consistently, overexpression of miR-497 significantly inhibited the mRNA (Figure 4D) and protein (Figure 4E) expression of FGFR1 in AGS and SGC-7901 cells compared with transfected with miR-NC. These results indicated that miR-497 directly suppressed FGFR1 expression by targeting its 3′-UTR.

### Overexpressed FGFR1 reversed miR-497-induced growth inhibition and apoptosis

**Figure 5.**
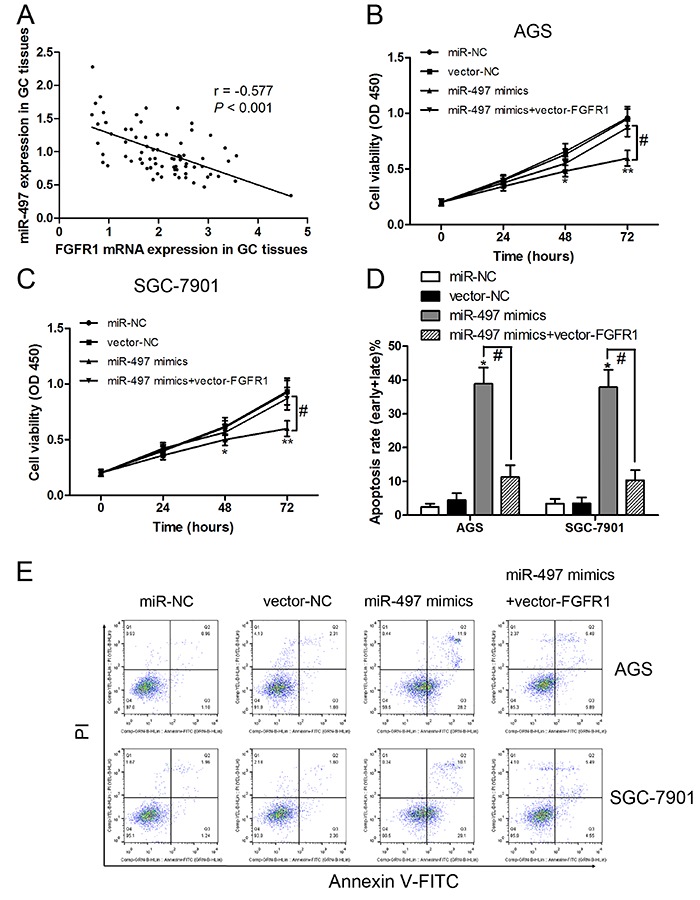
Correlation between miR-497 and fibroblast growth factor receptor 1 (FGFR1) by Spearman's rank correlation analysis (*A*). After transfection with miR-497 mimics or miR-497 mimics combined with vector-FGF1, cell viability was measured by CCK-8 assay (*B* and *C*), and cell apoptosis was detected by flow cytometry in AGS and SGC-7901 cells (*D* and *E*). Data are reported as means±SE. *P<0.05, **P<0.01; ^#^P<0.05 (ANOVA, n=3 in each group).

Based on the above results, we concluded that FGFR1 might act as an antagonist to miR-497 in gastric cancer cell proliferation and apoptosis. We also found that the expression levels of FGFR1 and miR-497 were inversely correlated in the 74 gastric cancer tissues (r=–0.577, P<0.001; Figure 5A). To further verify these conclusions, we performed a co-expression assay with miR-497 mimics and FGFR1 co-transfected into AGS and SGC-7901 cells. The results indicated that overexpression of FGFR1 reversed the growth inhibition (Figure 5B and C) and apoptosis (Figure 5D and E) of miR-497 mimics in AGS and SGC-7901 cells. These findings suggest that overexpression of miR-497 inhibited proliferation and induced apoptosis in gastric cancer through the suppression of FGFR1.

## Discussion

In our present study, FGFR1 was up-regulated in gastric cancer tissues and cell lines and associated with poor survival prognosis, metastasis, and TNM stage, suggesting that FGFR1 played a crucial role in the carcinogenesis of gastric cancer. As an oncogene, FGFR1 knockout mediated cell growth inhibition and apoptosis in gastric cancer cells. Importantly, we found a post-transcriptional regulatory mechanism of miR-497 as a tumor suppressor, which had been reported in many cancer types (17,20,30). miR-497 targeted to inhibition of FGFR1 expression blocked gastric cancer cells proliferation and induced apoptosis, indicating that miR-497 and FGFR1 may serve as a potential therapeutic target for the treatment of gastric cancer.

Emerging evidence has suggested that FGFR1 plays crucial roles in the pathological processes of gastric cancer, including cell proliferation, metastasis, recurrence, and poor prognosis (7,27,28). The prognostic significance of FGFR1 in gastric cancer was firstly reported by Murase et al. (27) suggesting that high expression of FGFR1 is associated with poor survival in patients with gastric cancer. Subsequently, further study shows that high expression of FGFR1 is associated with tumor progression and survival in diffuse-type gastric cancer, but not in intestinal-type gastric cancer (7). Consistent with those results, we observed that high expression of FGFR1 was also correlated with lymph node metastasis, recurrence, higher TNM stage, and poor overall survival. However, the comparative analysis of the expression of FGFR1 in gastric cancer tissues and adjacent non-tumor tissues or normal cells and gastric cancer cell lines had not been presented in these studies (7,27). In our study, we found that FGFR1 was significantly increased in gastric cancer tissues compared to adjacent non-tumor tissues. In addition, the expression of FGFR1 was up-regulated in gastric cancer cell lines compared to normal GES-1 cells. Based on these results, we hypothesized that FGFR1 may be an oncogene in the progression of gastric cancer. To verify this conclusion, sh-RNA was used to inhibit the expression of FGFR1 *in vitro*, and found that cell proliferation was inhibited and apoptosis was increased in AGS and SGC-7901 cells after knockout of FGFR1 by si-RNA. Similar results were reported in lung cancer (31), hepatocellular carcinoma (32), and prostate cancer (33). Therefore, we firmly believe that FGFR1 as an oncogene can serve as an independent prognostic factor and a very promising therapeutic target for patients with gastric cancer.

Another important finding was that miR-497, known as a tumor suppressor (18), directly targeted the 3′-UTR of FGFR1 to inhibit the process of translation, which is a classical post-transcriptional mechanism involved in cell growth inhibition and apoptosis in AGS and SGC-7901 cells. A previous study has demonstrated that miR-497 has a lower expression in gastric cancer tissues than non-tumorous tissues (34). In multidrug-resistant human gastric cancer cell line SGC7901, the expression of miR-497 is down-regulated (35). Similarly, Li et al. (22) also showed that miR-497 is decreased in both gastric cancer tissues and cell lines. Our study showed the same outcomes: the expression of miR-497 was inhibited in gastric cancer tissues and cell lines. *In vitro* and *in vivo* observations indicate that restoration of miR-497 can block the growth of gastric cancer cells (22). We also found that overexpressed miR-497 inhibited gastric cancer cells proliferation and induced apoptosis *in vitro*. Based on these findings, miR-497 may function as a tumor suppressor miRNA in gastric cancer.

Our study also found that FGFR1 was a direct target of miR-497, and we were the first to explore the functional roles of miR-497 and FGFR1 in gastric cancer cells proliferation and apoptosis. Overexpression of FGFR1 reversed miR-497 mimics inducing growth inhibition and apoptosis, as well as the expression levels of FGFR1 and miR-497 were inversely correlated in the tumor tissues. Thus, we proposed the possibility that FGFR1 and miR-497 had exactly the opposite role in the progression and development of gastric cancer.

Taken together, our results showed that miR-497 inhibited FGFR1 expression and consequently induced growth inhibition and apoptosis in gastric cancer cells. Experimental validations delineated a novel regulatory mechanism that miR-497 could serve as tumor suppressor in the progression of gastric cancer by blocking FGFR1 expression. More importantly, miR-497 and FGFR1 might contribute to the development of prognostic indices and therapeutic targets for patients with gastric cancer.
